# Nonlinear Ultrasonic C-Scan Imaging for Contact-Type Defects in Diffusion-Bonded Joints—A Case Study

**DOI:** 10.3390/ma17061288

**Published:** 2024-03-11

**Authors:** Chi Zhang, Qianghua Pan, Taili Liu, Lin Zhang, Tie Gang

**Affiliations:** 1 China Special Equipment Inspection and Research Institute, Beijing 100029, China; 2 State Key Laboratory of Advanced Welding and Joining, Harbin Institute of Technology, Harbin 150001, China

**Keywords:** diffusion bonding, contact-type defects, non-destructive testing, C-scan imaging, nonlinear ultrasound

## Abstract

Diffusion bonding technology is widely used in the connection of precision components, yet accurately and reliably detecting contact-type defects on the bond interface still remains a significant problem. Nonlinear ultrasonic methods have been proven to be sensitive to contact-type defects; however, the use of continuous wave or tone burst wave excitation limits its wider application. In this paper, dual-probe nonlinear ultrasonic testing with pulse wave excitation is proposed to detect contact-type defects in diffusion-bonded joints. A titanium alloy diffusion-bonded specimen with artificial defects was fabricated, and the corresponding detection device was designed based on the existing ultrasonic C-scan testing system. A C-scan imaging program based on nonlinear parameters was developed by extracting the fundamental and second harmonic waves of the reflection echo on the bond interface. The results demonstrated that the proposed detection scheme can obtain the nonlinear parameters of diffusion-bonded interfaces, and the nonlinear ultrasonic C-scan image of the bond interface is also obtained. The nonlinear parameter in the contact-type defects areas calculated from the bond interface echo is about 10 times (20 dB) higher than that in macro defects areas, whose gap is about 10 μm. The results indicate that the nonlinear ultrasonic methods seem to be more sensitive to contact-type defects and have a great potential to complement the insufficient detection capability of linear ultrasound for diffusion-bonded joints.

## 1. Introduction

Diffusion bonding (or diffusion welding) is a welding method that applies a certain temperature and pressure to two weldments to make the two surfaces come into contact with each other and maintains them for a certain time in a vacuum or protective atmosphere so that the contact surfaces undergo local microscopic plastic deformation and atomic diffusion to achieve connection [1,2]. It has become an indispensable technology in aerospace, power electronics, and other industries used in the manufacturing processes of engine blades and fuselage shells [3,4].

However, diffusion bonding has high requirements for welding processes such as temperature and pressure. The surface roughness, cleanliness, and material composition and microstructure of the welding surface can also have a significant impact on the welding process, and defects such as incomplete welding are prone to occur at the weld interface. Of particular note is the contact-type defect that may occur in diffusion-bonded joints, the nature of which has not been clearly studied, and it often represents the lack of welding at the two interfaces. Since the surfaces of the defect are in close contact, conventional ultrasound is almost completely transmitted at the interface, making it difficult to detect [5]. The lack of accurate detection methods for contact-type defects in diffusion-bonded interface seriously affects the large-scale application of diffusion-bonded structures in important fields such as aerospace, and therefore, a reliable non-destructive testing method is urgently needed to evaluate the quality of diffusion bonding.

Initially, interface resistance measurement was used for the non-destructive testing of diffusion-bonded joints [6], but it is generally applicable to the detection of larger-sized defects. Considering the similarity between diffusion-bonded joints and brazed or resistance spot welded joints, the ultrasound C-scan method has been used to scan the diffusion-bonded interface and process the obtained images to roughly obtain the interface bonding ratio, so as to optimize the diffusion bonding process [7], or estimate the mechanical properties (such as impact toughness) of diffusion-bonded joints based on the interface bonding ratio [8]. However, the longitudinal resolution of the C-scan method is limited by the pulse width of the transmitted signal of the probe. If the detection capability of small defects is to be enhanced, the most direct method is to increase the detection frequency of the probe [9,10], but the attenuation of sound waves in the medium also increases sharply, that is, the detection thickness decreases. At the same time, increasing the detection frequency also makes the detection system more complex and increases the detection cost. Another way is to analyze the full waveform of the C-scan to extract waveforms, spectra, phase characteristics, etc. that reflect the performance of the diffusion-bonded interface, that is, using the ultrasonic characteristic scanning method (F-scan) to detect the diffusion-bonded components [11,12], thereby further improving their detection capability. However, this method requires complex signal processing, and it still falls within the scope of linear ultrasound, and its detection sensitivity will not exceed 1/2 of the corresponding wavelength.

During the formation of diffusion-bonded joints, if a large number of micro-pores, micro-cracks, and other defects occur at the interface, the mechanical properties of the interface will exhibit high nonlinear characteristics. When the incident sound wave reaches this interface, it will undergo a series of nonlinear interactions with it, and significant nonlinear components may appear in the received signal [13,14]. Therefore, people consider using nonlinear acoustic methods to characterize the interface state. This method is theoretically not limited by the detection frequency and has higher detection sensitivity to small defects, especially when contact-type defects exist, and significant nonlinear components will appear in the received signal [15,16]. Studies have shown that linear ultrasound is more sensitive to macro-sized defects, and for contact-type defects, the linear ultrasound echo is weak, but the nonlinear response is strong [17,18,19]. However, there are currently few studies on the use of nonlinear ultrasound methods in pulse-echo mode to detect contact-type defects in diffusion-bonded joints. If the nonlinear component can be excited by an ultrasound pulse and extracted from the echo, the nonlinear ultrasonic method can be applied to practical testing. Furthermore, like the linear ultrasonic C-scan method, the nonlinear ultrasonic C-scan image can be obtained.

In this paper, the nonlinear ultrasonic testing in pulse mode is investigated and the nonlinear ultrasonic C-scan imaging is developed. A diffusion bonding sample with artificial cracks on the bond interface is designed and fabricated, a nonlinear ultrasonic C-scan scheme using dual-transducers and the corresponding device are also designed, and then the sample is detected with nonlinear ultrasonic imaging and compared with linear ultrasonic C-scan; finally, the metallography results of the bond joint is used to verify the detection method.

## 2. Principle of Nonlinear Ultrasonic Testing

The high-order harmonic method in nonlinear acoustic testing methods is used in this paper. Generally, there are two physical mechanisms for generating high-order harmonics: the nonlinear elastic mechanism (nonlinear elasticity), also known as classical nonlinearity, and the contact nonlinearity mechanism, also known as non-classical nonlinearity [20].

Classical nonlinearity theory is generally used to explain material nonlinear phenomena caused by plastic deformation, fatigue damage, etc. It believes that the stress–strain (*σ-ε*) relationship in the material damage area shows a nonlinear relationship, that is,

(1) $$\sigma =E\varepsilon \left(1+\gamma \varepsilon +\cdots \right),$$

where *E* is Young’s modulus; *γ* is the second-order nonlinear elastic coefficient, also known as the nonlinear parameter.

Thus, the nonlinear wave equation can be obtained (only one-dimensional situation is given in this paper, taking the first-order approximation)

(2) $$\rho \frac{\partial^{2}u}{\partial t^{2}}=E\frac{\partial^{2}u}{\partial x^{2}}+2E\gamma \frac{\partial u}{\partial x}\frac{\partial^{2}u}{\partial x^{2}},$$

where *ρ* is the density of the medium; *u* is the displacement of the mass point; *t* is the propagation time; *x* is the propagation distance.

Under the conditions of constant wave number *k* and propagation distance *x*, if the amplitude values *A*_1_ and *A*_2_ of the fundamental wave and the second harmonic signal at a specific position are known, the formula for calculating the nonlinear parameter can be obtained as follows:

(3) $$\gamma =\frac{8A_{2}}{k^{2}xA_{1}^{2}},$$

According to the measured nonlinear parameters, the degree of material damage can be characterized.

**Figure 1:**
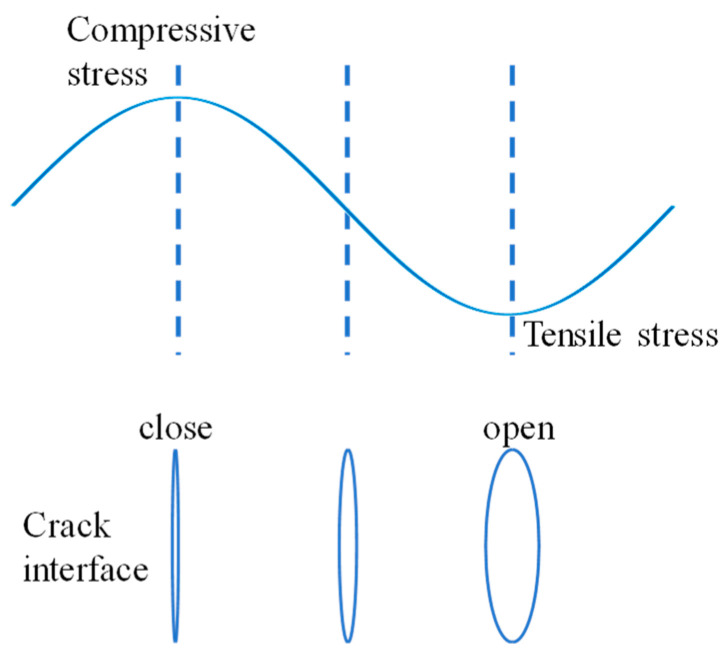
Mechanism of contact nonlinearity [20].

However, when cracks and other contact defects appear in the structure, their acoustic nonlinearity will increase sharply, and the nonlinear parameter *γ* value measured at this time will be much larger than before the appearance of the crack. Its mechanism can be represented phenomenologically by Figure 1. When the ultrasonic wave is evident on the contact defect interface, the two contact surfaces produce relative motion under the action of the ultrasound. In a complete vibration cycle, the ultrasonic wave generates compressive stress and tensile stress on the interface, and the interface closes and opens under its action; in the closed state, it is equivalent to an intact interface, and the ultrasound passes through almost completely, whereas in the open state, it is equivalent to the interface between the medium and air, and the sound wave is almost completely reflected at this interface. That is, only half a cycle of the sound wave can transmit through or reflect from the interface and then be received. Therefore, the higher harmonic components are present in the received signal, and the nonlinear parameter can be calculated from the received signal.

According to the above theory, the nonlinear detection method is more sensitive to small defects, especially the contact-type defects in diffusion-bonded or adhesive-bonded joints than the linear methods.

## 3. Materials and Methods

### 3.1. Ultrasonic C-Scan System

In this experiment, a conventional water-immersed focused ultrasonic C-scan device was modified to achieve nonlinear ultrasonic C-scan testing. The principle of ultrasonic C-scan technology is to use a computer-controlled ultrasonic transducer (probe) to scan the workpiece line by line, and continuously display the reflected wave amplitude at a specific position inside the workpiece in the form of a grayscale image (or pseudo-color image) after sampling and quantization, thereby providing a cross-sectional image of the internal part of the workpiece within the corresponding detection range. The instrument used in this paper is an ultrasonic C-scan system from Physical Acoustics Corporation, West Windsor Township, NJ, USA, and the main performance parameters are shown in Table 1. To improve the experimental accuracy, two point-focused probes were used, and the specific parameters are shown in Table 2.

### 3.2. Diffusion-Bonded Sample Fabrication

The material used in this experiment is titanium alloy (TA15), and its components are shown in Table 3.

**Figure 2:**
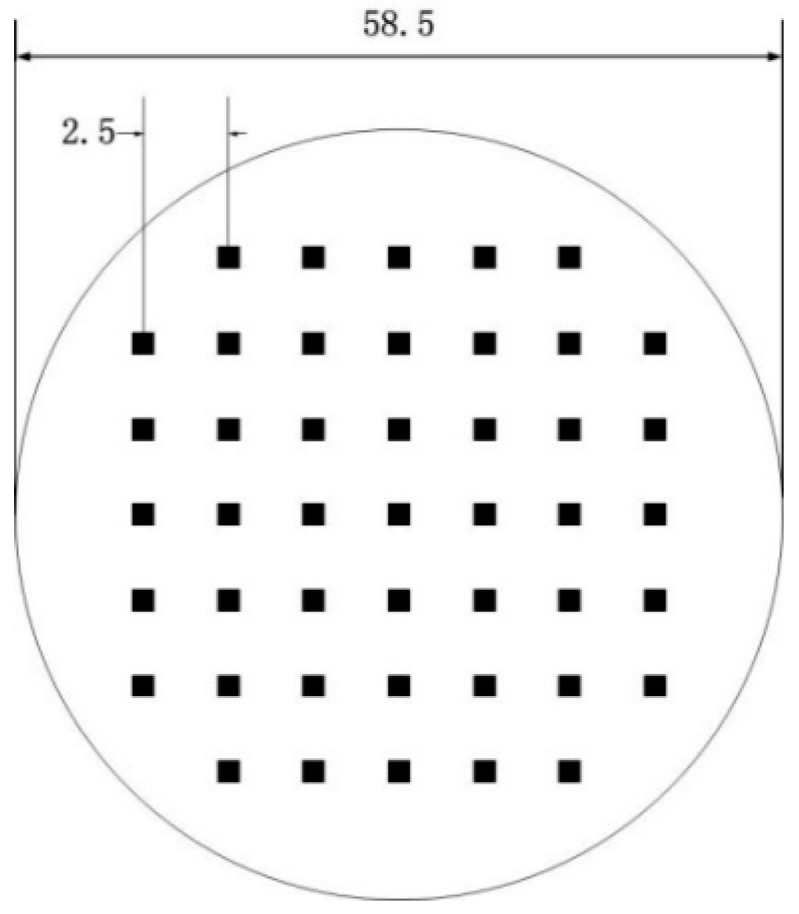
Schematic diagram of specimen.

The specimen is fabricated with two circular plates, and the diameter and thickness of the plates are 58.5 mm and 2 mm, respectively. Two pieces of plates are stacked and heated under pressure for diffusion bonding in a vacuum furnace. The bonding parameters are shown in Table 4. By applying the required shape and size of the solder resist to the interface and using the normal process for welding, the region coated with the solder resist is not bonded, while the region without the solder resist is expected to form a good weld seam. The shape of the defects in the specimens is square, with a side length of 1.2 mm and a defect spacing of 2.5 mm, as shown in Figure 2. Due to the influence of high pressure and high temperature during the bonding, the actual diameter of the sample is about 60 mm.

### 3.3. Nonlinear Ultrasonic Testing Scheme

**Figure 3:**
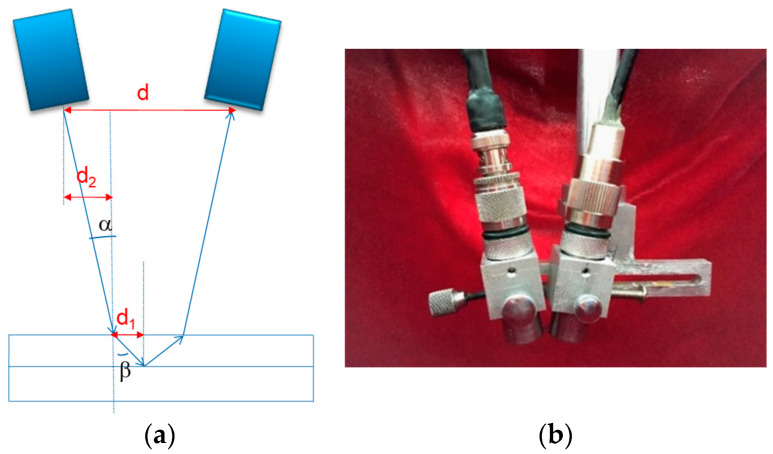
Dual-probe nonlinear testing scheme and assembly diagram: (**a**) dual-probe nonlinear testing scheme; (**b**) probe assembly diagram.

For the diffusion-bonded sample structure being tested, a dual-probe detection scheme is designed. The signal excitation system generates a fundamental wave signal with a frequency of f, which is transformed into the corresponding sound wave by the transducer. After being reflected by the workpiece, the presence and amplitude of the second harmonic in the received detection signal are evaluated to assess the quality of the joint. Therefore, the center frequency of the receiving transducer should be 2f. The assembly diagram of the probes is shown in Figure 3.

### 3.4. Detection Parameter Settings

To achieve detection using longitudinal waves, it is necessary to determine the incident angle α and the distance between the two probes (d in Figure 3). Considering that more ultrasonic energy is concentrated at the joint, the incident angle of the sound wave on the water/workpiece interface should be smaller than the first critical angle of this interface to avoid total reflection by the longitudinal wave. That is,

(4) $$\sin\alpha <\frac{c_{W}}{c_{T}}=\frac{1480}{5900},$$

where *c*_W_ and *c*_T_ are the acoustic wave speed in water and TA15 alloy, respectively. The incident angle α should be less than 14.5° according to the calculation. *α* is set to 12°, and the refracted angle *β* can be obtained from the following equation

(5) $$\sin\beta =\frac{5900\sin\alpha }{1480}$$

Therefore, the probe center distance d = 2 × (d1 + d2) = 16 mm.

### 3.5. Harmonic Extraction Program

**Figure 4:**
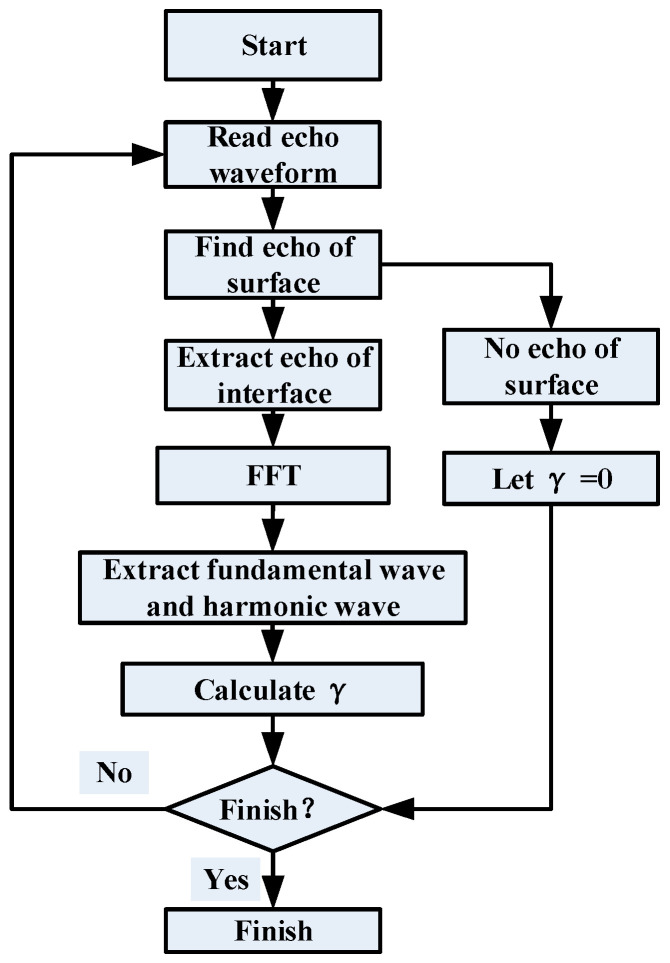
Flowchart of nonlinear ultrasonic C-scan detection.

The receiving probe is connected to the ultrasonic C-scan system receiving end, and the received signal is collected and stored by the scan system’s built-in data acquisition card (Type: AD-IPR-1210; Manufacturer: Physical Acoustics Corporation, USA; Sampling rate: 100 MSa/s; Resolution: 12 bit). In order to obtain an obvious interface echo on the crack for obtaining a clear linear C-scan image and for extracting the second harmonic easily, the testing parameters of linear ultrasonic C-scan were optimized as shown in Table 5. The C-scan system can generate an image of the sample using a built-in program during the scan process; the amplitude of the bonding interface echo was calculated from the time domain, and each amplitude value was filled into a pixel corresponding to the detection position to form the C-scan image. However, due to the strong excitation signal and the weak nonlinearity of the interface, the received signal contains not only the desired harmonic component but also a strong fundamental component. What is more, the amplitude of the fundamental component may be much higher than that of the harmonic. Therefore, the scanned image generated directly by the system’s built-in program is not necessarily the desired nonlinear testing result. It is actually a linear ultrasonic C-scan testing result. To obtain a nonlinear ultrasonic C-scan image, the waveform of each scan point is extracted from the linear C-scan data, and then the echo of the bond interface is separated from the waveform and transferred to the frequency domain using a fast Fourier transform (FFT); next, the amplitudes of the fundamental component (A1) and harmonic component (A2) are obtained from the frequency domain, and finally, the nonlinear parameter γ of each scan position is calculated using Equation (3) to form the nonlinear ultrasonic C-scan image. The flowchart is shown in Figure 4.

## 4. Results

**Figure 5:**
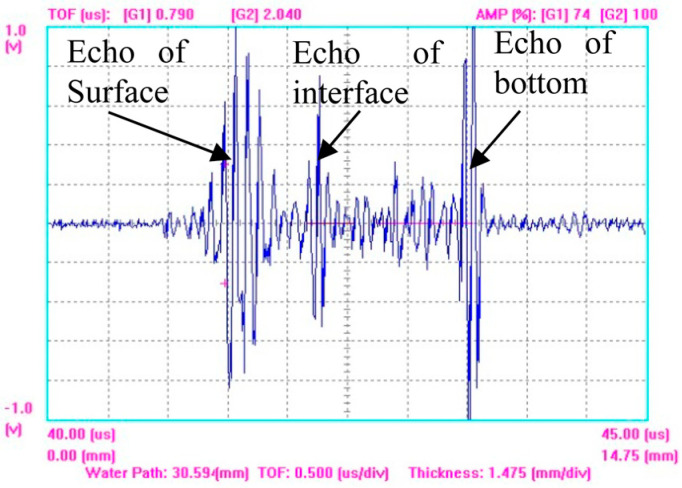
Typical detection echoes of diffusion bonding interface.

According to the calculation results in 3.4, the probe spacing and focusing positions were adjusted, and typical received echoes at the diffusion bonding specimen interface were obtained, as shown in Figure 5. The arrows in the figure indicate the surface reflection echoes of the specimen, the diffusion bonding interface reflection echoes, and the bottom reflection echoes of the specimen.

**Figure 6:**
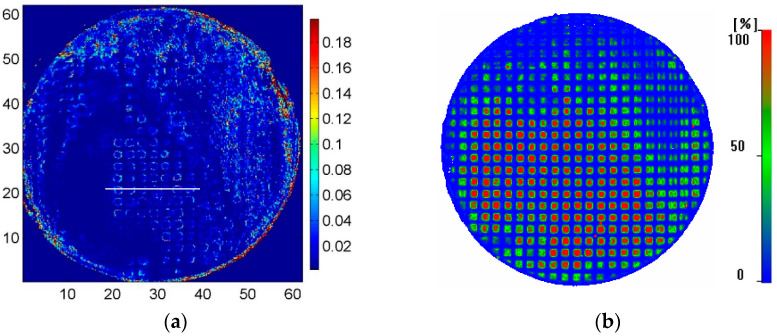
Nonlinear and linear ultrasonic C-scan images: (**a**) nonlinear ultrasonic C-scan; (**b**) linear ultrasonic C-scan.

From Figure 5, it can be seen that under the conditions of oblique incident reception by dual probes, there are strong reflection echoes at the defect interface. The receiving probe used in the experiment has a center frequency of 20 MHz. Theoretically, the received signal only contains the 20 MHz signal and its nearby frequencies. However, in actual detection, due to the strong excitation signal, weak harmonic signals may be received even though the center frequency of the receiving probe is 20 MHz, which masks the weak second harmonic component. Therefore, to obtain the nonlinear testing result, it is necessary to calculate the nonlinear parameter *γ* based on the scanning signal and then regenerate the image corresponding to the detection position. Since the C-scan system used in the experiment does not have the function of harmonic extraction, the extraction program in 3.4 is used to complete the above signal processing and image generation process. That is, the interface waveform at each detection point is separately extracted and transformed into the frequency domain, and the amplitudes of the fundamental wave and the second harmonic are measured separately, so as to calculate the nonlinear parameter of this point interface. The obtained nonlinear C-scan imaging results are shown in Figure 6a, where the brighter the color level, the higher the value of the nonlinear parameter. It can be observed that the lower part of the specimen shows low nonlinear parameters, while the upper and right parts show high nonlinear parameter values. The central region of the detection image appears as a hollow square, indicating that the central region of the square has a low nonlinear coefficient, while the edge region has a high nonlinear coefficient. According to the nonlinear detection principle, it is inferred that there are a large number of contact-type defects in the upper and right parts of the specimen, the central region of the artificial defect in the central region of the specimen is completely unbonded, and there is contact between the upper and lower interfaces in the edge region, forming a contact-type defect. Under the action of sound waves, the contact-type defect region will undergo opening and closing movements, thereby changing the waveform of the reflected sound wave; that is, the current detection point has a high nonlinear parameter value, which is reflected in the detection image as a high color level.

For comparison, Figure 6b shows the results of conventional linear ultrasound C-scanning, using a single probe in pulse-echo mode. The probe used is the B probe specified in Table 2, with a center frequency of 20 MHz. The probe is adjusted to focus the sound beam at the interface of the specimen. From the image, it can be observed that the upper and right parts of the specimen show irregular shape arrays with lower gray levels, while the middle, left, and lower parts display regular square dot arrays with higher gray levels in the central region and lower gray levels in the surrounding areas. This indicates that the artificial defects in the upper and left parts have poorer fabrication quality, with possible partial contact between the upper and lower interfaces of the defects, resulting in lower sound reflection coefficients. On the other hand, the artificial defects in the middle, left, and lower parts have better fabrication quality, with their positions and shapes matching the expected ones. This may be due to uneven pressure during the welding process. The sound waves are almost entirely reflected back without penetrating through the defects.

It can also be noticed in Figure 6b that for the C-scan results of most square defects, the grey levels in the middle part are higher than those of the edges. This indicates that there is some contact at the edges of the defects in the middle part, resulting in lower sound reflection coefficients at the edges. It is speculated that this is due to the larger size of the square defects fabricated artificially, with the largest gap in the center and smaller gaps towards the edges, until complete contact and fusion. According to the principles of nonlinear ultrasound testing, this technique is more sensitive to small defects and defects with close contact, where the amplitude of the second harmonic at the intact region between the defect center and the defect should be lower, while the amplitude at the defect edge should be higher.

By comparing the nonlinear and linear detection results shown in Figure 6a,b, it can be observed that they complement each other well. For the well-welded samples and samples with large defect gaps in the left part, both nonlinear detection results show low echo signals, while the conventional linear ultrasound can detect regularly arranged artificial defects. For the upper and right parts of the samples, the nonlinear detection results show high echo signals, while the conventional linear ultrasound detection shows smaller echo amplitudes. Especially in the middle region, the nonlinear ultrasound detection shows a hollow square, while the linear ultrasound detection results show a high echo reflection coefficient at the center of the square. Therefore, in practical detection, the results of both methods should be combined to make a reasonable judgment on the quality of the detection area.

**Figure 7:**
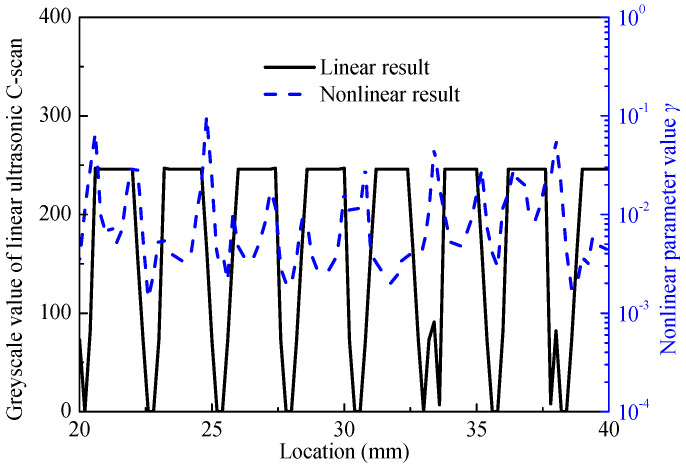
Comparison of linear and nonlinear ultrasonic scan results.

To further study the relationship between the nonlinear scan and linear ultrasound scan detection results, the grayscale distribution at the same position in the middle region (indicated by the white line in Figure 6a) was obtained and compared, as shown in Figure 7. It can be observed that the areas with higher nonlinear parameters are mostly located at the edges of defects detected by linear ultrasound, indicating the presence of contact-type defects with strong nonlinear effects in this area, while the center region of the defects has relatively lower nonlinear parameters, which is consistent with the principle of nonlinear detection. The nonlinear parameter (γ) values in the edge area (~0.003) of the defects are on average 10 times (20 dB) higher than that in the center (~0.03) of the defects.

**Figure 8:**
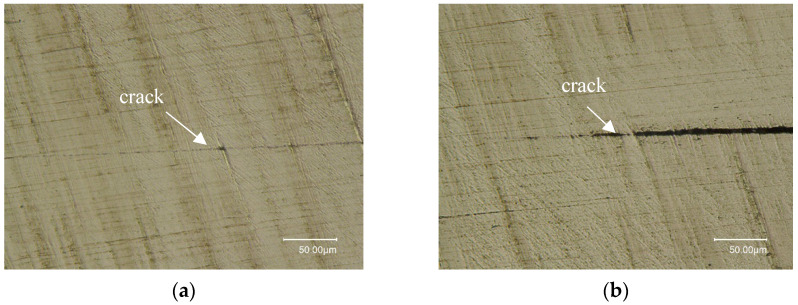
Metallographic dissection results of the sample: (**a**) crack interface of the right part; (**b**) crack interface of the middle part.

To validate the above analysis, the sample is dissected with a wire-electrode cutting process; the cross-section is perpendicular to the bond interface and traverses a row of artificial cracks. The cross-section plane was ground with sandpaper from coarse type to fine type and then polished with polishing cloth and suspension. Figure 8 shows the dissected results of the defects in the right and middle parts of the sample. It can be observed that the defects in the right part fit tightly, while the defects in the middle part have obvious gaps (about 5 μm), but the gaps become smaller as they approach the edges, and even complete contact and fusion can be seen.

## 5. Discussion

Next, the above results are compared with traditional ultrasonic C-scan results of diffusion bonds and nonlinear ultrasonic testing using tone burst wave excitation. According to the detection theory, when there is a large gap, the linear ultrasound reflection coefficient is high, while the nonlinear parameter is low. Contact-type defects have high nonlinear parameters and low linear sound wave reflection coefficients. The dissected results indicate that the nonlinear parameter imaging results in this study are consistent with the theoretical expectations. In Ref [11], according to the principle of acoustic echo phase change on the crack interface, the researchers proposed a new algorithm using both amplitude and phase characteristics of the echo on the bond interface. The unbond defect and kissing bond (contact-type defect) in the diffusion bond joint have been detected by the method. However, it requires extracting both amplitude and phasing characteristics during detection. For most nonlinear ultrasonic methods, including that in Ref [15,16,17,18,19], the excitation signal is a tone burst wave, which makes it difficult to extract the echo on the bond interface. Compared with the previous methods, the nonlinear ultrasonic C-scan imaging proposed in this work can easily obtain the echo on the bond interface and only needs simple signal processing to extract the nonlinear parameter and to form the C-can image. Over and above, the nonlinear ultrasound detection method has a high sensitivity to contact-type defects in diffusion-welded joint interfaces. By using nonlinear parameter imaging for C-scan detection of diffusion-welded samples, it can effectively complement the deficiencies of linear ultrasound methods in detecting such defects. It is suggested that both methods be combined to achieve a more accurate evaluation of the quality of diffusion-welded joints.

## 6. Conclusions

In this study, the dual-probe nonlinear ultrasound C-scan method in pulse mode is proposed and its effectiveness in detecting contact-type defects is verified. A device is designed and fabricated for pulse wave excitation and reception. The results demonstrate that the contact-type defects in the titanium alloy diffusion-welded joints exhibit strong nonlinear effects. The nonlinear parameter on the bond interface is determined by extracting the fundamental and second harmonic wave from the interface echo, and the results demonstrate that the amplitude of the nonlinear parameter on the contact-type defects is about 10 times higher than that of the macro-cracks. The nonlinear ultrasonic C-scan image is achieved based on nonlinear parameters, and the result indicates that it can provide a powerful complement to linear ultrasound detection.

According to the results, it is suggested that the combination of the two methods is expected to achieve an accurate evaluation of the quality of diffusion-welded joints. In this work, the dual-probe mode was used due to the lack of a broad bandwidth transducer. In the future, the transducer with broad bandwidth can be developed to excite a pulse wave and receive the second harmonic of the echo, and then the nonlinear ultrasonic imaging can be more widely applied to the field test.

## Figures and Tables

**Table 1:** Performance arameters of Ulturasonic C-scan system.

Maximum Scanning Speed (mm/s)	Scanning Range (mm)	Scanning Step Size (mm)	Maximum Gain (dB)	Maximum Sampling Rate (M/s)	Motion Axes	Maximum Scanning Speed (mm/s)
250	600 × 450 × 300	0.01	60	100	3	250

**Table 2:** Probe Performance Parameters.

Probe	Center Frequency (MHz)	Focus Distance (mm)	Focus Column Diameter (mm)	Focus Column Length (mm)	Element Diameter (mm)
A	10	38.1	0.44	5.5	12.5
B	20	38.1	0.44	10.7	6.25

**Table 3:** Components of the TA15.

Ti	Al	Zr	Mo	V	Impurity Elements
88.4%	6.4%	1.9%	1.3%	1.8%	0.2%

**Table 4:** Diffusion bonding parameters.

Temperature (°C)	Holding Time (h)	Pressure (MPa)	Weld Surface Roughness
940	2	2	Ra0.4

**Table 5:** Optimized ultrasonic C-scan parameters.

Pulse Voltage (V)	Pulse Width (MHz)	Receiving Gain (dB)	Receiving Filter Bandwidth (MHz)
400	25	0	0.5–30 (pass band)

## Data Availability

The data presented in this study are available on reasonable request from the corresponding author. Due to the nature of this research, participants of this study did not agree for their data to be shared publicly.
